# Ultrasound-guided peripheral nerve blocks for preoperative pain management in hip fractures: a systematic review

**DOI:** 10.1186/s12871-022-01720-7

**Published:** 2022-06-21

**Authors:** Oskar Wilborg Exsteen, Christine Nygaard Svendsen, Christian Rothe, Kai Henrik Wiborg Lange, Lars Hyldborg Lundstrøm

**Affiliations:** 1 Department of Anaesthesiology and Intensive Care, Copenhagen University Hospital, Nordsjællands Hospital, Hillerød, Denmark; 2 Department of Clinical Medicine, University of Copenhagen, Copenhagen, Denmark

**Keywords:** Regional anaesthesia, Peripheral nerve block, Hip fracture, Ultrasonography

## Abstract

Systematic reviews associate peripheral nerve blocks based on anatomic landmarks or nerve stimulation with reduced pain and need for systemic analgesia in hip fracture patients. We aimed to investigate the effect of ultrasound-guided nerve blocks compared to conventional analgesia for preoperative pain management in hip fractures. Five databases were searched until June 2021 to identify randomised controlled trials. Two independent authors extracted data and assessed risk of bias. Data was pooled for meta-analysis and quality of evidence was evaluated using Grades of Recommendation Assessment, Development and Evaluation (GRADE). We included 12 trials (976 participants) comparing ultrasound-guided nerve blocks to conventional systemic analgesia. In favour of ultrasound, pain measured closest to two hours after block placement decreased with a mean difference of -2.26 (VAS 0 to 10); (*p *< 0.001) 95% CI [–2.97 to –1.55]. In favour of ultrasound, preoperative analgesic usage of iv. morphine equivalents in milligram decreased with a mean difference of –5.34 (*p*=0.003) 95% CI [–8.11 to –2.58]. Time from admission until surgery ranged from six hours to more than three days. Further, ultrasound-guided nerve blocks may be associated with a lower frequency of delirium: risk ratio 0.6 (*p* = 0.03) 95% CI [0.38 to 0.94], fewer serious adverse events: risk ratio 0.33 (*p* = 0.006) 95% CI [0.15 to 0.73] and higher patient satisfaction: mean difference 25.9 (VAS 0 to 100) (*p* < 0.001) 95% CI [19.74 to 32.07]. However, the quality of evidence was judged low or very low. In conclusion, despite low quality of evidence, ultrasound-guided blocks were associated with benefits compared to conventional systemic analgesia.

**Supplementary Information:**

The online version contains supplementary material available at 10.1186/s12871-022-01720-7.

## Introduction

Hip fractures are a serious health problem. They are most common in the frail, elderly population and are associated with considerable pain in the perioperative stage. In addition to subjective discomfort, untreated pain may lead to increased risk of complications and delirium in this patient group [1, 2]. Effective pain therapy is challenging, especially in this frail population with significant comorbidities. Conventional treatment with opioids and NSAIDs is associated with typical side effects and peripheral nerve blocks (PNBs) may not be effective because of the many nerves involved in pain transmission from the fractured area.

A recent Cochrane review concluded that PNBs performed perioperatively reduce pain on movement within 30 minutes after block placement, risk of acute confusional state and probably also reduce the risk of chest infection and time to first mobilisation [3]. Likewise, other systematic reviews focusing on specific PNBs like the fascia iliaca compartment block and the femoral nerve block demonstrated pain reduction and reduced opioid consumption [4, 5]. However, in these reviews the majority of included randomised controlled trials used PNBs without ultrasound (US)-guidance, i.e. they only used anatomic landmarks or nerve stimulation for guidance. It seems intuitive that using US-guidance should be more effective than using a blind technique, since it allows a trained physician to deposit the local anaesthetic (LA) with much more precision.

In this systematic review we therefore aimed to compare the analgesic effects of US-guided PNBs (US-PNBs) to conventional pain management with systemic use of analgesics. We hypothesised that US-PNBs reduce pain and opioid consumption prior to surgery compared to conventional pain management.

## Methods

The protocol for this systematic review was registered with PROSPERO, (International Prospective Register of Systematic Reviews, CRD42021239510). It is presented according to the PRISMA statement [6]. We included randomised controlled trials (RCTs) in adult patients (≥ 18 years) undergoing surgery following a fracture to the proximal femur. Unpublished trials were eligible if trial data and methodological descriptions could be provided either in written form or through direct contact with the authors. Trials using quasi-randomisation and observational studies were excluded. As stated in our protocol, we planned to compare the analgesic effects of US-PNBs to conventional pain management and to PNBs performed using only nerve stimulation, anatomic landmarks or both, respectively. However, in our search we did not find trials comparing US-PNBs to PNBs performed without US-guidance. Thus, in this review we focus on the comparison between US-PNBs and systemic analgesia.

We included trials comparing single shot or continuous (catheter based) US-PNBs to conventional pain management (systemic use of opioids, NSAID/paracetamol) with or without a sham block (injection of saline) The intervention, i.e. the US-PNB, had to be administered before surgery and therefore all trials comparing PNBs given during or after surgery were excluded. Only the following PNBs were included: femoral nerve block, fascia iliaca compartment block (both superior and inferior to the inguinal ligament) and 3-in-1 block. Trials using the term 3-in-1 block were categorised as femoral nerve block in our assessment. We included trials where the US-PNB was performed with only US-guidance or combined with nerve stimulation (dual-guidance).

We searched MEDLINE (Pubmed), Embase (OVID), Cochrane Central Register of Controlled Trials (CENTRAL) in the Cochrane Library, CINAHL and International Web of Science until June 16^th^, 2021. The full search string, developed for MEDLINE using MeSH terms and keywords related to two concepts; hip fractures and nerve blocks, is presented in the supplementary material (Additional file 2). The search was not limited to contain “ultrasonography” as a concept to ensure that we would not omit any trials that failed to mention block technique in the title or abstract, neither did we limit the initial search to trials registered as randomised controlled trials. Additionally, a hand search of bibliographic references and citations of the studies that met our inclusion criteria as well as the relevant systematic reviews was conducted to ensure as high a saturation as possible. Only trials reported in Latin alphabets were included. We used Covidence systematic review software (Veritas Health Innovation, Melbourne, Australia, 2021) for data management [7]. In the process of selecting trials, two authors (OE and one of CS; CR; KL or LHL) independently screened the titles and abstracts yielded by the search and excluded based on our eligibility criteria. Two authors (OE and one of CS; CR; KL or LHL) then examined the full-text reports and extracted data on a predefined standardised paper form. Any disagreements between the two authors were resolved by discussion and - if necessary - a final decision was made by a third author (LHL or KL).

### Primary outcome

Primary outcome was pain after block placement measured before surgery. We used preoperative pain scores as either visual analogue scale (VAS) score or numeric rating scale (NRS) score closest to two hours after intervention to minimise the range of time points and clinical heterogeneity. If pain scores were reported both at rest and at movement, we would include pain at movement in our assessments.

### Secondary outcomes

Secondary outcomes were: 1) Opioid analgesic usage before surgery (measured as iv. morphine equivalents); 2: Time to first request for additional analgesia; 3: Prevalence of serious adverse events. We defined serious adverse events according to the International Conference on Harmonisation Guidelines (ICH 1995) as: "any event that leads to death, is life-threatening, requires in-patient hospitalisation or prolongation of existing hospitalisation, results in persistent or significant disability, and any important medical event, which may jeopardise the patient or requires intervention to prevent it" [8]. All other adverse events were considered non-serious; 4: Patient satisfaction. The definitions of patient satisfaction presented in the individual articles were accepted; 5: Prevalence and severity of delirium. The definitions of delirium presented in the individual articles were accepted; 6: Length of hospitalisation/length of stay; 7: Mortality. We used the longest follow-up data from each trial.

We evaluated the validity and design characteristics of each trial by evaluating the trials for major sources of bias. Two authors independently used the risk of bias approach described in the Cochrane Handbook for Systematic Reviews of Interventions as a tool for assessing risk of bias in the included trials [9]. The following risk of bias domains were assessed: allocation sequence generation, allocation concealment, blinding of participants and investigators, blinding of outcome assessment, incomplete outcome data, selective outcome reporting and other bias like sponsor bias. These seven domains were judged to be either high risk, low risk or unclear and the trial was deemed to be in overall high risk of bias if one or more of the domains were high risk.

### Statistical analysis

We used Review Manager (RevMan 5.4) software to conduct all statistical analyses following the guidelines set out by the Cochrane Handbook [10]. We calculated the risk ratio (RR) with 95% confidence intervals (CI) for dichotomous variables (binary outcomes) and calculated mean difference with 95% CI for continuous outcomes. The primary outcome ‘pain after block placement’ was continuous, but different scoring scales might have been used. In case of non-convertible scoring scales, we calculated the standardised mean difference (SMD). If trials reported median with corresponding ranges/interquartile ranges, the values were converted into mean with standard deviation for our meta-analyses [11]. We planned the following sensitivity analysis of our primary outcome: Evaluation of the impact of trials with high or uncertain risk of bias versus trials with low risk of bias. Further, we planned the following subgroup analysis: Use of US-PNBs versus conventional pain treatment with or without sham block (comparisons of subgroups of femoral nerve block vs fascia iliaca compartment block).

The degree of heterogeneity observed in the results was quantified using an inconsistency factor (I^2^) statistic, which can be interpreted as the proportion of the total variation observed between the trials that is attributable to differences between trials rather than sampling error (chance) [12]. The I^2^ statistic suggests thresholds for low (25% to 49%), moderate (50% to 74%) and high (≥75%) heterogeneity [13]. We used the Chi^2^ test to provide an indication of heterogeneity between studies, with *P* ≤ 0.10 considered statistically significant.

Meta-analysis was visualised by a forest plot showing point estimates of mean and 95% CI. If I^2^ = 0, we would report the results from the fixed-effect model. In the case of I^2^ > 0, we would report the results from the random-effects model.

### Grade

We used the GRADE system to evaluate quality of evidence for specific outcomes [14]. The quality of evidence considers: (1) within study risk of bias (methodological quality); (2) directness of evidence; (3) heterogeneity of data; (4) precision of effect estimates; and (5) risk of publication bias. In GRADE there are four levels of certainty of evidence: Very low (the true effect is probably markedly different from the estimated effect); Low (the true effect might be markedly different from the estimated effect); Moderate (the true effect is probably close to the estimated effect); High (the true effect is probably similar to the estimated effect).

## Results

### Study selection

We identified 3056 references of which 942 were duplicates. Thus, 2114 study abstracts were screened for eligibility. Full text screening was performed in 118 of these studies and after this procedure 15 studies were found to fulfil the inclusion criteria [15–29]. However, three trials were subsequently excluded; one trial was only published as a detailed abstract and the author did not respond to our inquiry of supplementary data [24]; one study had to be excluded due to the participants in the intervention group not being randomised directly to US-PNBs [25] and one trial [26] permitted a single shot US-guided femoral nerve block as rescue treatment in the standard care (control). Most common reasons for exclusion at full text screening were no US-guidance, not RCT or wrong comparator.

### Study characteristics

Of the 12 included trials a total of 509 participants were randomised to receive a US-PNB (intervention) and 467 participants were randomised to control groups. Table 1 provides an overview of the characteristics of the included trials. The trials were published from 2010 to 2021 and the number of randomised participants ranged from 20 to 198. Seven trials investigated the femoral nerve block [15, 16, 18, 20, 21, 23, 28] and six investigated the fascia iliaca compartment block [17–19, 22, 27, 29]. Of the 12 trials, four sought to blind the patients by using a type of sham block [15, 17, 19, 20]. Eight trials had a single shot US-PNB as intervention [15–18, 20, 23, 27, 29] whereas four trials administered an initial bolus before placing a catheter for continuous infusion [19, 21, 22, 28]. One study described testing for block success [21]. One study used dual guidance [28]. Two trials compared more than one intervention to control; one had two parallel intervention groups (femoral nerve block and fascia iliaca compartment block) compared to one control group [18]; one trial included peridural anaesthesia, which was of no interest to this review [21]. We contacted seven authors in an attempt to collect additional data but received no responses [17–19, 23, 24, 27, 28]. In one case we included a study by collecting published information from conference abstract as well as unpublished data reported on ClinicalTrials.gov [18]. Control groups all used systemic analgesia, but drug, administration and dosage varied. Among the trials reporting the preoperative duration from block performance until surgery the time to surgery was more than 24 hours in the majority of the cases (Table 1).

**Table 1:** Characteristics of included trials comparing US-PNBs to systemic analgesia

			**Intervention**			**Control**				
Author (year)	N	Site	Type of PNB	LA type and dose	Time from admission to surgery	Analgesic treatment		Sham- block		Time from admission to surgery
Beaudoin [15] (2013)	36	USA	US-guided single shot FNB	25 ml 0.5% bupivacaine	480 (324-670) min (median, range)	iv. morphine		3 ml saline sc.		510 (341-704) min (median, range)
Beaupre [16] (2020)	73	Canada	US-guided single shot FNB	10 ml 1% ropivacaine + 5 ml 0.25% bupivacaine	29.8 (14.3) hours	Unspecified standard care treatment		None		26.8 (12.3) hours
Diakomi [17] (2020)	198	Greece	US-guided single shot FICB	40 ml 0.5% ropivacaine	No data	iv. 0.5 µg.kg^-1^ fentanyl as rescue		2 ml saline in inguinal region		No data
Dickman [18] (2010)	64	USA	US-guided single shot FNB or US-guided single shot FICB	30 ml 0.25% bupivacaine	No data	One dose of iv. 0.1 mg.kg^-1^ morphine sulphate		None		No data
Hao [19] (2019)	90	China	US-guided continuous FICB	30 ml 0.45% ropivacaine	29.49 (4.58) hours	0.05 mg im. fentanyl at VAS ≥ 5		Sham catheter (continuous saline infusion)		29.81 (3.78) hours
Jang [20] (2018)	34	South Korea	US-guided single shot FNB	0.3 ml.kg^-1^ up to 20 ml 0.5% bupivacaine	Elective surgery after 48 hours	iv. tramadol		3 ml saline sc.		Elective surgery after 48 hours
Luger [21] (2012)	20	Austria	US-guided continuous FNB	30 ml 0.25% bupivacaine	26.8 (18.6) hours	iv. piritramide and PCM		None		24.9 (1.7) hours
Ma [22] (2018)	88	China	US-guided continuous FICB	50 ml 0.4% ropivacaine	3.27 (0.82) days	po. tramadol and PCM		None		3.34 (0.89) days
Morrison [23] (2016)	161	USA	US-guided single shot FNB	20 ml 0.5% bupivacaine	1.5 (0-4) days (mean, range)	po. and iv. standard care (opioids and PCM)		None		1.4 (0-7) days (mean, range)
Thompson [27] (2020)	47	USA	US-guided single shot FICB	30 ml 0.25% ropivacaine	No data	PCM, tramadol or morphine according to VAS		None		No data
Uysal [28] (2020)	110	Turkey	Dual-guided continuous FNB	10 ml 0.25% bupivacaine	No data	iv. 15 mg.kg^-1^ PCM, 0.5 mg.kg^-1^ tramadol as rescue		None		No data
Yamamoto [29] (2019)	53	Japan	US-guided single shot FICB	40 ml 0.25% levobupivacaine	No data	iv. 1000 mg PCM at 6 and 12 hours post-op		None		No data

Data is presented as mean (SD) unless else is stated

*N* number of randomised participants, *PNB* peripheral nerve block, *US-guided* ultrasound-guided, *FNB* femoral nerve block, *FICB* fascia iliaca compartment block, *LA* local anaesthetic, *PCM* paracetamol, *iv.* intravenous, *sc.* subcutaneous, *im.* intramuscular, *po.* peroral, *VAS* visual analogue scale, *post-op* postoperatively

### Risk of bias

In our risk of bias assessment none of the trials were judged to have low risk of bias in all domains (Table 2). In our assessment of potential reporting bias, the funnel plot of our primary outcome did not express asymmetry, thus not indicating risk of bias. Of notice, three of the four trials that used a sham block [15, 17, 20] used smaller volumes of saline compared to the volumes of LA used in the intervention groups. Hereby the participants may be blinded, but the investigators performing the block were most likely not blinded. In our assessment, we categorised these trials with high risk of bias in the domain evaluating blinding of the investigators.

**Table 2:**
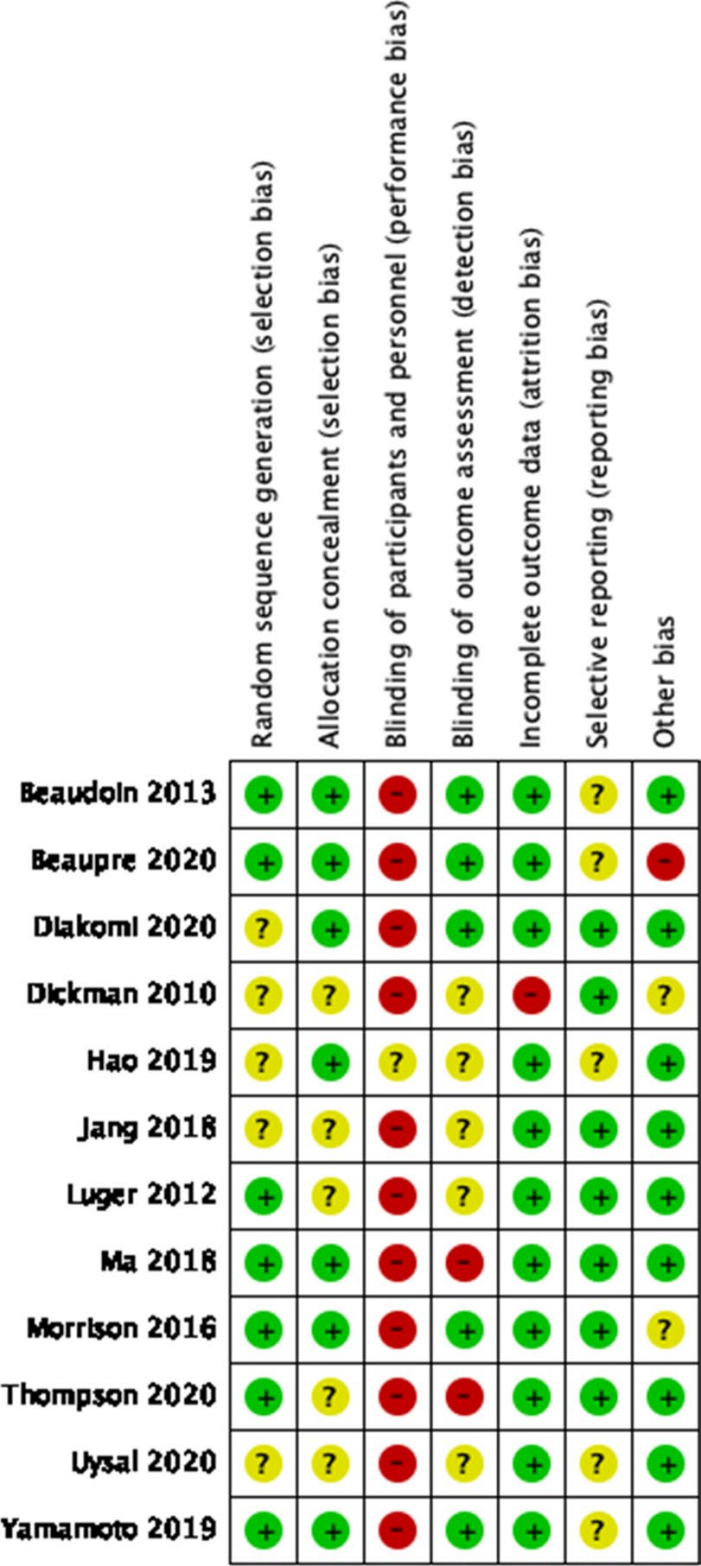
Risk of bias assessment

Cochrane Collaboration risk of bias summary: evaluation of bias risk items for each included study. Green circle denotes low risk of bias; yellow circle, unclear risk of bias; red circle, high risk of bias

### Effects of intervention

Main results are shown in Table 3.

**Table 3:** Summary of findings including GRADE assessment [30]

**US-guided peripheral nerve blocks compared to conventional analgesia for preoperative pain management in hip fractures**						
**Patient or population**: Patients with hip fractures; **Setting**: Preoperative pain management; **Intervention**: US-guided peripheral nerve block; **Comparison**: Conventional pain management with systemic use of analgesics.						
Outcomes	**Anticipated absolute effects**^**a**^ (95% CI)		Relative effect (95% CI)	№ of participants (studies)	Certainty of the evidence (GRADE)	Comments
	**Risk with conventional pain management**	**Risk with US-guided peripheral nerve block**				
Pain after block placement (VAS 0 to 10 (worse))	The mean pain after block placement ranged from **4.0 to 7.10**	MD **2.26 cm lower** (1.55 lower to 2.97 lower)	-	561 (8 RCTs)	⨁⨁◯◯ LOW	Downgraded two points due to poor methodological quality (high performance bias) and statistical and clinical heterogeneity (inconsistency).
Additional analgesic usage (iv. morphine equivalents (mg))	The mean additional analgesic usage ranged from **2.55-14** mg iv. ME	MD **5.34 mg iv. ME lower** (8.11 lower to 2.58 lower)	-	173 (4 RCTs)	⨁⨁◯◯ LOW	Downgraded two points due to poor methodological quality (high performance bias) and statistical and clinical heterogeneity (inconsistency).
Prevalence of serious adverse events	16 per 100	**5 per 100** (2 to 12)	**RR 0.33** (0.15 to 0.73)	277 (3 RCTs)	⨁◯◯◯ VERY LOW	Downgraded three points due to poor methodological quality (high performance bias), clinical heterogeneity (inconsistency) and few trials in analysis with trials reporting zero-zero events not being included (imprecision)
Length of stay (Days)	The mean length of stay ranged from **6-14** Days	MD **0.92 Days lower** (3.55 lower to 1.71 higher)	-	423 (3 RCTs)	⨁◯◯◯ VERY LOW	Downgraded three points due to poor methodological quality (high performance bias), statistical and clinical heterogeneity (inconsistency) and few trials with meta-analysis showing wide confidence interval (imprecision)
Patient satisfaction (VAS 0 to 100 (best))	The mean patient satisfaction ranged from **45-72**	MD **25.91 higher** (19.74 higher to 32.07 higher)	-	135 (2 RCTs)	⨁◯◯◯ VERY LOW	Downgraded three points due to poor methodological quality (high performance bias), statistical heterogeneity (inconsistency) and few trials with few participants (imprecision).
Prevalence of delirium	23 per 100	**14 per 100** (8 to 23)	**RR 0.60** (0.38 to 0.94)	382 (4 RCTs)	⨁◯◯◯ VERY LOW	Downgraded three points due to poor methodological quality (high performance bias), clinical heterogeneity (inconsistency) and few trials in analysis (imprecision).
Mortality (Longest follow up)	4 per 100	**4 per 100** (2 to 9)	**RR 1.00** (0.41 to 2.40)	470 (4 RCTs)	⨁◯◯◯ VERY LOW	Downgraded three points due to poor methodological quality (high performance bias), clinical heterogeneity (inconsistency) and wide confidence intervals due to few events with zero-zero events excluded (imprecision).

GRADE Working Group grades of evidence: The quality considers: (1) within study risk of bias (methodological quality); (2) the directness of the evidence; (3) heterogeneity of the data; (4) precision of effect estimates; and (5) risk of publication bias

High certainty: We are very confident that the true effect lies close to that of the estimate of the effect

Moderate certainty: We are moderately confident in the effect estimate: The true effect is likely to be close to the estimate of the effect, but there is a possibility that it is substantially different

Low certainty: Our confidence in the effect estimate is limited: The true effect may be substantially different from the estimate of the effect

Very low certainty: We have very little confidence in the effect estimate: The true effect is likely to be substantially different from the estimate of effect

*CI* Confidence interval, *MD* Mean difference, *RR* Risk ratio, *ME* Morphine equivalent, *RCT* Randomised controlled trial, VAS Visual analogue scale

^a^The risk in the intervention group (and its 95% confidence interval) is based on the assumed risk in the comparison group and the **relative effect** of the intervention (and its 95% CI)

### Primary outcome

#### Pain after block placement

Eight trials reported at least one pain score obtained between intervention and surgery with time of measurement ranging from 15 minutes to 48 hours [15, 18–23, 28]. Two trials [21, 22] provided pain scores at movement and six trials did not specify the circumstances of measurements [15, 18–20, 23, 28]. Data are presented in Table 4. In addition to the eight trials, one trial [17] claimed to have measured significantly lower NRS scores “both prior and after positioning for spinal anaesthesia” in the intervention group, but they only reported data on postoperative pain and the authors did not respond to our inquiry of preoperative data. The study was therefore not included in our meta-analysis of the primary outcome.

**Table 4:** Results of individual studies reporting on pain after block placement

**Author (year)**	Intervention	Control	Pain after block placement (VAS)			Additional analgesic usage (mg iv. morphine equivalents)	
			Time of measurement	US-PNB	Control	US-PNB	Control
Beaudoin [15] (2013)^a^	US single shot FNB with 25 ml bupivacaine 5 mg.ml^-1^	iv. morphine	Baseline 15 min 1 h **2 h** 4 h	7.19 (1.4) 4.16 (1.53) 3.43 (1.32) **3.63 (1.87)** 4.28 (1.61)	7.97 (3.01) 7.17 (2.55) 7.48 (2.46) **7.1 (2.63)** 7.99 (3.01)	0.0 (2.0 – 6.0), median (range)	5.0 (2.0 – 21.0), median (range)
Dickman [18] (2010)	US single shot FNB with 30 ml 0.25% bupivacaine	iv. 0.1 mg.kg^-1^ morphine sulphate	Baseline 30 min 1 h **2 h** 4 h 8 h	5.17 (3.29) 1.94 (2.43) 2.58 (3.06) **2.65 (2.49)** 3.15 (2.70) 3.20 (2.28)	6.98 (1.87) 5.13 (2.70) 4.40 (2.92) **4.00 (2.98)** 4.83 (2.58) 3.74 (2.89)	-	-
	US single shot FICB with 30 ml 0.25% bupivacaine		Baseline 30 min 1 h **2 h** 4 h 8 h	5.50 (3.99) 2.05 (2.61) 1.90 (2.38) **1.30 (1.89)** 1.72 (1.98) 2.35 (3.07)	6.98 (1.87) 5.13 (2.70) 4.40 (2.92) **4.00 (2.98)** 4.83 (2.58) 3.74 (2.89)		
Hao [19] (2019)	US continuous FICB with 30 ml 0.45% ropivacaine	0.05 mg im. fentanyl at VAS ≥ 5	Baseline **2 h** 4 h	7.81 (0.79) **2.74 (0.73)** 2.23 (0.43)	8.07 (0.64) **4.19 (0.40)** 3.67 (0.34)	4 (10.5)	14 (6.5)
Jang [20] (2018)^a^	US single shot FNB with 0.3 ml.kg^-1^ up to 20 ml 0.5% bupivacaine	iv. tramadol	Baseline **4 h** 24 h 48 h	7.1 (0.79) **3.62 (0.67)** 4.5 (0.63) 5.11 (0.73)	6.8 (0.81) **7.06 (0.57)** 5.75 (0.67) 5.18 (0.6)	1.25 (0.912)	5.37 (3.77)
Luger [21] (2012)^a^	US continuous FNB with 30 ml 0.25% bupivacaine	iv. piritramide and PCM	During rest			0.56 (1.8)	2.55 (3.83)
			Baseline 1 h 12 h	6.66 (1.15) 0.56 (0.27) 0.21 (0.19)	6.34 (1.2) 4.47 (1.74) 1.31 (0.64)		
			During movement				
			Baseline **1 h** 12 h	8.64 (0.57) **2.45 (0.66)** 2.11 (0.92)	8.74 (0.54) **6.19 (1.13)** 4.04 (1.25)		
Ma [22] (2019)^a^	US continuous FICB with 50 ml 0.4% ropivacaine	po. tramadol (50 mg) and PCM (500 mg)	During rest			-	-
			Baseline 1 h	4.27 (0.96) 2.13 (0.69)	4.58 (1.09) 2.32 (0.8)		
			Passive movement				
			Baseline **1 h**	7.16 (1.18) **3.33 (0.89)**	7.12 (1.3) **4.85 (1.07)**		
Morrison [23] (2016)	US single shot FNB with 20 ml 0.5% bupivacaine	po. and iv. standard care (opioids and PCM)	1 h **2 h**	3.7 (3.1) **3.5 (3.1)**	5.3 (3.2) **5.3 (3.2)**	-	-
Uysal [28] (2020)	Dual-guided continuous FNB with 10 ml 0.25% bupivacaine	iv. 15 mg.kg^-1^ PCM (0.5 mg.kg^-1^ tramadol as rescue)	**4 h**	**3.32 (0.92)**	**4.47 (1.06)**	-	-

Pain after block placement (reported in cm VAS) and additional analgesic usage (in iv. morphine equivalents). Data is presented as mean (SD) unless else is stated. Numbers in bold text were used for meta-analysis.

Four included studies did not report on our primary outcome and are thus not mentioned in this table [16, 17, 27, 29]

*US-PNB* ultrasound guided peripheral nerve block, *FNB* femoral nerve block, *FICB* fascia iliaca compartment block, *iv.* intravenous, *im.* intramuscular; *po.* peroral, *VAS* visual analogue scale, *PCM* paracetamol; *min* minutes; *h* hours.

^a^VAS-scores were extracted from graphical presentation (mean, SD). Conversion factor (CF) for fentanyl: 50x, CF for tramadol: 0.1x, CF for piritramide: 0.75x.

**Fig. 1:**
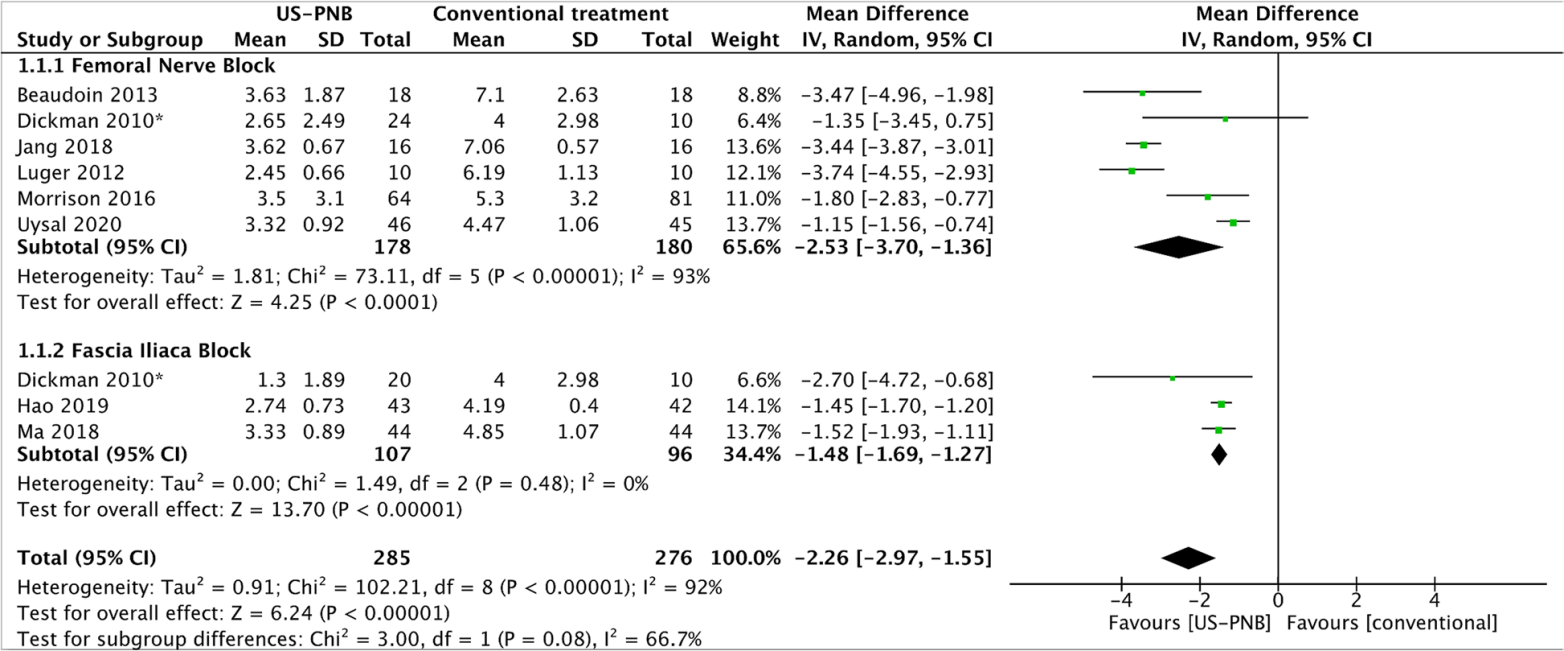
Pain after block placement or corresponding time in control group. Forest plot of pain reduction after preoperative ultrasound guided peripheral nerve blocks compared to systemic analgesia in hip fracture patients. Mean and SD are presented at 10 cm visual analogue scale (VAS). US-PNB, ultrasound-guided peripheral nerve block.*due to the two intervention groups, the number of participants in the control group has been split in two equal groups in order to perform subgroup analysis of femoral nerve block and fascia iliaca compartment block.

In the eight trials reporting on preoperative pain scores, 285 participants were allocated to a US-PNB (intervention) and 276 participants were allocated to conventional analgesia (control). Our meta-analysis (Fig. 1), using the VAS/NRS score (0-10) for pain measurement demonstrated a significant pain reduction when using US-PNBs (random effects model, mean difference –2.26; *p* < 0.001; 95% CI –2.97 to –1.55; I^2^ = 92%; GRADE = low). All trials were judged with high risk of bias, thus we performed no sensitivity analysis comparing trials with low vs high risk of bias. We performed a subgroup analysis comparing the subgroups of femoral nerve block to fascia iliaca compartment block (Fig. 1). We found a mean difference in VAS (0 to 10) of –2.53 with femoral nerve block compared to conventional treatment with or without sham block and a mean difference of –1.48 with fascia iliaca compartment block compared to conventional treatment with or without sham block. A test for subgroup differences showed a non-significant (*p* = 0.08) difference between femoral nerve block and fascia iliaca compartment block regarding effect of intervention on preoperative pain.

### Secondary outcomes

#### Opioid analgesic usage (iv. morphine equivalents)

**Fig. 2:**
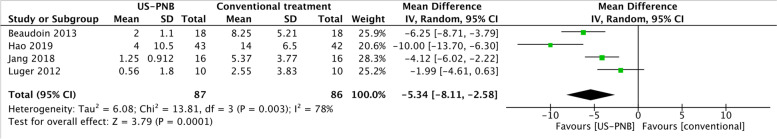
Opioid analgesic usage (i.v. morphine equivalents). Forest plot of preoperative additional opioid usage after preoperative ultrasound guided peripheral nerve blocks compared to systemic analgesia in hip fracture patients. Mean and SD are presented as iv. morphine equivalents in mg. US-PNB, ultrasound-guided peripheral nerve block.

Six trials reported on preoperative additional analgesic usage [15, 16, 19–21, 23], but two studies [16, 23] were left out of meta-analysis due to data not being convertible or suitable for meta-analysis using standardised mean difference. The trials used different types of analgesics and we therefore calculated opioid equivalents of the preoperative consumption (Table 4) [31]. Our meta-analysis (Fig. 2) showed a significant opioid sparing effect (iv. morphine equivalents in mg) of US-PNBs compared to conventional pain treatment (random effects model; mean difference –5.34; *p* = 0.003; 95% CI –8.11 to –2.58; I^2^ = 78%; GRADE = low).

#### Time to first request for additional analgesia

No trials measured preoperative time to first request of analgesics.

#### Prevalence of serious adverse effects/adverse events

Seven studies [15, 17, 18, 22, 23, 27, 29] mentioned monitoring for serious adverse events or complications related to nerve blocks. No trials reported any complications in direct relation to the PNB (e.g. hematoma/vessel puncture, nerve damage, infection or local anaesthetic systemic toxicity). One study specifically reported on incidence of cardiovascular, pulmonary or cerebral complications [22] while another mentioned monitoring for severe opioid-related side effects [23]. Three trials [15, 22, 23] registered one or more events. Trials that found zero events in each category were left out of meta-analysis, as RevMan 5.4 is not able to analyse zero-zero events. Our meta-analysis (Supplementary Fig. 1) found a significantly reduced risk of experiencing an SAE with US-PNBs compared to conventional analgesia: (fixed effects model; RR 0.33; *p* = 0.006; 95% CI 0.15 to 0.73; I^2^ = 0%; GRADE = very low).

#### Patient satisfaction

Two trials reported on patient satisfaction with one using a 0-100 VAS satisfaction score [22] and one using a 0-25 scale [27]. VAS 100 was considered the highest possible patient satisfaction and we therefore multiplied the latter scale by four for our meta-analysis. Both trials favoured US-PNBs over control in our meta-analysis (Supplementary Fig. 2) (random effects model; mean difference 25.91; *p* < 0.001; 95% CI 19.74 to 32.07; I^2^ = 76%; GRADE: very low) .

#### Prevalence and severity of delirium

The occurrence of delirium was a subject of investigation in four trials [19, 23, 28, 29]. Three trials used the Confusion Assessment Method (CAM) [19, 23, 29] while one used the Delirium Rating Scale-R-98 (DRS-R-98) [28]. CAM is used to detect the presence of delirium while DRS-R-98 provides a score of severity. In the study using DRS-R-98, the severity score was provided preoperatively, but not postoperatively. It was not clear if the intervention had been performed before obtaining the score and severity of delirium was therefore not included in our analysis. All trials reported fewer cases of delirium when patients received a US-PNB compared to systemic analgesia with one trial showing a statistically significant reduction [19]. Meta-analysis (Supplementary Fig. 3), (fixed effects model; RR 0.60, *p* = 0.03; 95% CI 0.38 to 0.94; I^2^ = 0%; GRADE: very low).

#### Length of stay

Three studies [17, 22, 23] measured length of stay (LOS). Mean LOS varied greatly between trials and LOS was similar between intervention and control groups. Meta-analysis (Supplementary Fig. 4) found no significant difference (random effects model; mean difference –0.92 days; *p* = 0.49; 95% CI –3.55 to 1.71; I^2^ = 86%; GRADE = very low)**.**

#### Mortality

Four studies [17, 19, 22, 28] reported at least one death during stay or at follow-up. However, among the remaining trials, it was not clear if there were any follow-up on vital status of the patients.

One trial [17] had the longest follow up at six months whereas the other three [19, 22, 28] only reported mortalities during hospitalisation. Our meta-analysis (Supplementary Fig. 5) found no significant difference between US-PNBs and conventional analgesia (fixed effects model; RR of 1.00; *p* = 0.99; 95% CI 0.41 to 2.40; I^2^ = 0%; GRADE = very low).

## Discussion

### Summary of evidence

Our review suggests that among patients suffering from a hip fracture, a preoperative US-PNB is associated with a significant pain reduction and reduced need for systemic analgesics compared to conventional analgesia. Our results may also indicate a lower risk of delirium, SAE and higher patient satisfaction in patients receiving a US-PNB. Our findings should be interpreted in the light of the quality of evidence of these results, which ranged from low to very low.

With reservations to the reduced quality of evidence, our results indicate an approximal mean pain reduction of VAS at 2.25 cm to an anticipated absolute effect ranging from VAS 1.6 to 3 with a reduction in iv. morphine consumption of 5 mg. At first, the pain relief seems clinically relevant, however, as the pain score was evaluated close to two hours after performing the block, it is not possible to evaluate the effect of the block during the prolonged time from performing the block until start of surgery. The opioid sparing effect seems small and may be of less clinical importance. However, the potential for higher patient satisfaction and reduction in SAEs and delirium may be of clinical importance. A recent study [32] has shown no significant difference in incidences of postoperative delirium in hip fracture patients when using spinal/epidural anaesthesia vs general anaesthesia. Thus, the potential reduction in incidences of delirium when applying a PNB could therefore be of clinical importance as well.

Our review adds to the already existing body of evidence supporting the use of peripheral nerve blocks for preoperative pain management in hip fractures. We reviewed US-PNBs as this is considered the gold standard in anaesthesia today [33] and because of the lack of systematic reviews regarding this subject. The literature has shown that there is evidence to support use of anatomical PNBs over systemic analgesia. Some may argue that the US-guided technique is easy to perform and more reliable than the conventional PNB technique. However, our results on pain reduction and decrease in opioid consumption were comparable with the findings in the reviews [4, 5] comparing conventional PNB techniques with conventional pain management. We did not identify trials evaluating whether US-PNBs reduce pain and opioid consumption prior to surgery compared to other PNB-techniques without US-guidance. We acknowledge that there are situations where knowledge and expertise in landmark-based techniques can be meaningful. As an example, recent feasibility studies have investigated the use of PNBs in pre-hospital care, performed by paramedics or nurses at the scene of the accident, showing significant pain reduction and high patient satisfaction [34, 35].

A major problem in many studies investigating peripheral nerve blocks for hip fractures is that block success is not tested. Reasons include impeded testing of involved dermatomes (e.g. due to dementia) and myotomes (because of fracture). A successful nerve block does not necessarily result in a reduction in pain, but a failed block will most likely not reduce pain. This is of special importance when performing blocks with low success rates (technically difficult blocks) and will have major impact on the measured outcomes. Moreover, there will be considerable confounding when not all nerves from the affected regions are blocked. This applies to blocks for the hip and knee region as well as for truncal blocks.

Studies of recent years have shown that US-guidance is not used as often as one might think. An audit in the UK regarding the use of nerve blocks for femoral fractures showed that 74% of emergency departments had access to US-guidance, but 46% of emergency departments gave nerve blocks blindly and only 10% used US-guidance regularly for femoral nerve blocks [36]. An observational trend study of national data in the US showed that of patients receiving a PNB for hip arthroplasty, only 3.2% were performed using US-guidance [37]. Reasons for low use of US-guidance could be lack in training, equipment being unavailable or lack of evidence regarding the beneficial value over usual procedures.

### Limitations and quality of evidence

The limitations of the included trials were mostly related to the risk of bias due to lack of blinding, high degree of statistical heterogeneity and some degree of clinical heterogeneity. Further, for some outcomes the number of trials and participants were limited, thus there was a high degree of imprecision, hereby indicating a high risk of random error. Despite our relatively narrow inclusion criteria, our review was limited to some degree because of clinical heterogeneity. The included trials varied in several areas like block performance, type of LA, analgesics in control group, type of rescue analgesics and time of outcome measurement. We intended to perform meta-regression analysis in cases of statistical heterogeneity. According to the Cochrane Handbook, meta-regression should generally not be considered when the meta-analysis contains fewer than 10 studies and thus meta-regression was not performed in our analyses [10].

It is of high relevance to investigate adverse events. The screening for adverse events was complicated by the fact that the trials used different definitions. Often, events such as PONV, hypotension and desaturation were reported, but total number of patients experiencing an adverse event was never stated. We have used a confirmatory approach to investigate adverse events, where the aim is to synthesize data on pre-specified adverse events. A key limitation of the confirmatory approach is the inability to handle unanticipated adverse effects that are reported in the included studies [38]. We chose to investigate SAE and complications related to PNBs. This outcome has limitations since we miss all the patients that experience classic side effects to opioids which we hypothesise can be avoided using PNBs. However, we should be able to report if PNBs result in considerable risk of damage at the injection site or other serious events. We did not include trials with zero-zero events in our meta-analysis, which may therefore overestimate the prevalence of SAEs and mortality.

The included trials used different types of LA (either bupivacaine, ropivacaine or levobupivacaine). They are all considered to produce a nerve blocking effect within 30-60 min and nerve block duration of 6-12 hours depending on the application site [39–41]. It therefore seemed plausible to compare these LAs in the same analysis and to compare the time of measuring pain at 1, 2 and 4 hours as equal. However, the time between block performance and surgery in the majority of the trials was more than 24 hours, exceeding the expected block duration. Regional anaesthesia and systemic analgesia are not competing, but complementary methods. Thus, systemic analgesia reduces rebound pain in the event of decreasing block effect or catheter dislocation [42, 43] and may have impact on more of the outcomes other than our primary outcome. It is a further limitation that the patients in the PNB groups may also have received systemic analgesics pre- and intrahospitally before the block placement, which may have impacted outcomes measured after block performance. However, we have no detailed data available from the included trials concerning this.

Blinding can be difficult when examining PNBs. Ideally, for a double blinded study, the performing physician would inject the same volume, either LA or saline, without knowing what was injected, but this is often deemed unethical because of the unnecessary risk of nerve damage or vessel puncture. Of the included trials performing a sham block, three trials [15, 17, 20] injected 2-3 ml saline either subcutaneously or in the inguinal region. While the intent is to blind both the treating physician and the patient, it is hard to argue that this type of pseudo-blinding with such a low volume blinds the physician. One trial [19] placed a catheter for continuous saline infusion, which could be acknowledged as true blinding of both patient and personnel. However, it was not clearly stated if the physician placing the catheter was blinded to the administration of the initial bolus of LA/saline.

Our subgroup analysis did not find a statistically significant difference between femoral nerve block and fascia iliaca compartment block concerning our primary outcome. However, the analysis showed a trend towards the femoral nerve block being more effective. This finding should be interpreted with caution, as the number of trials and participants in the fascia iliaca compartment block group was limited, thus proposing a substantial risk of random error.

## Conclusion

This review supports the use of US guided preoperative nerve blocks for hip fractures, suggesting reduced pain, lower need of opioids and reduced risk of delirium and SAEs compared to systemic analgesia. The evidence was, however, low due to lack of blinding and statistical and clinical heterogeneity.

## Supplementary Information

**Additional file 1: Supplementary Figure 1. **Prevalence of serious adverse events. **Supplementary Figure 2.** Patient satisfaction. **Supplementary Figure 3.** Prevalence of delirium. **Supplementary Figure 4.** Length of stay. **Supplementary Figure 5.** Mortality.

**Additional file 2.** Search strategy.

## Data Availability

The datasets used and/or analysed during the current study are available from the corresponding author on reasonable request.

## Abbreviations

PNB: Peripheral nerve block
US-PNB: Ultrasound-guided peripheral nerve block
RCT: Randomised controlled trial
LA: Local anaesthetic
VAS: Visual analogue scale
NRS: Numeric rating scale
RR: Risk ratio
CI: Confidence interval
SMD: Standardised mean difference
SAE: Serious adverse event
LOS: Length of stay
PONV: Postoperative nausea and vomiting
