# Supplementary material for: Ultrasound-guided peripheral nerve blocks for preoperative pain management in hip fractures: a systematic review
Source: BMC Anesthesiol. 2022 Jun 21;22:192. doi: 10.1186/s12871-022-01720-7 (PMC9210678; doi:10.1186/s12871-022-01720-7)
Supplement: Supplementary file 1 — Additional file 1: Supplementary Figure 1. Prevalence of serious adverse events. Supplementary Figure 2. Patient satisfaction. Supplementary Figure 3. Prevalence of delirium. Supplementary Figure 4. Length of stay. Supplementary Figure 5. Mortality. [file 12871_2022_1720_MOESM1_ESM.docx]

**Supplementary material**

**Supplementary Figure 1. Prevalence of serious adverse events**

**
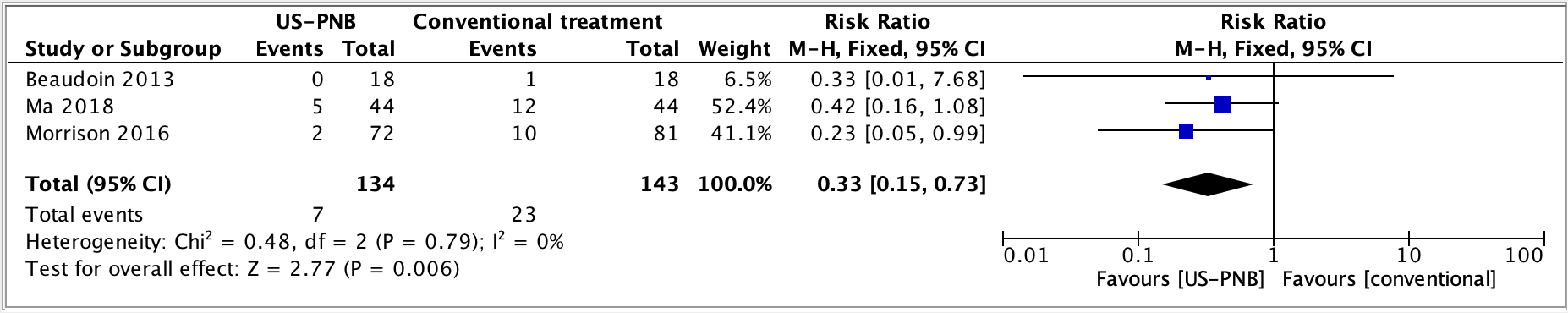
**

*Forest plot of serious adverse events after preoperative ultrasound guided peripheral nerve blocks compared to systemic analgesia in hip fracture patients.*

*US-PNB, ultrasound-guided peripheral nerve block.*

**Supplementary Figure 2. Patient satisfaction**

**
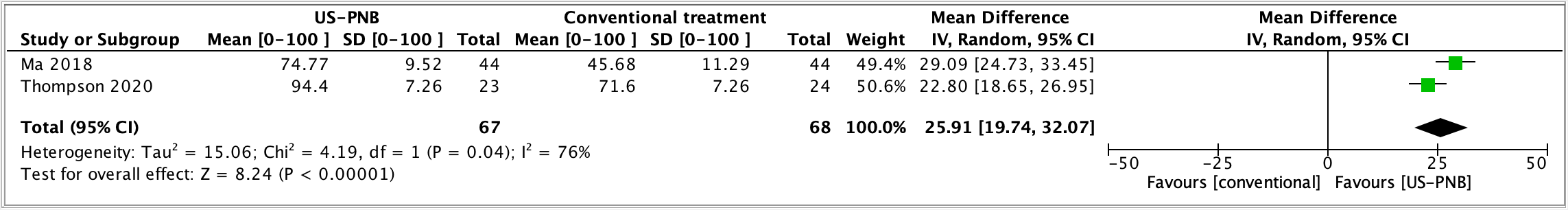
**

*Forest plot of patient satisfaction after preoperative ultrasound guided peripheral nerve blocks compared to systemic analgesia in hip fracture patients. Mean and SD are presented at 0-100 scale, 100 being the highest satisfaction.*

*US-PNB, ultrasound-guided peripheral nerve block.*

**Supplementary Figure 3. Prevalence of delirium**


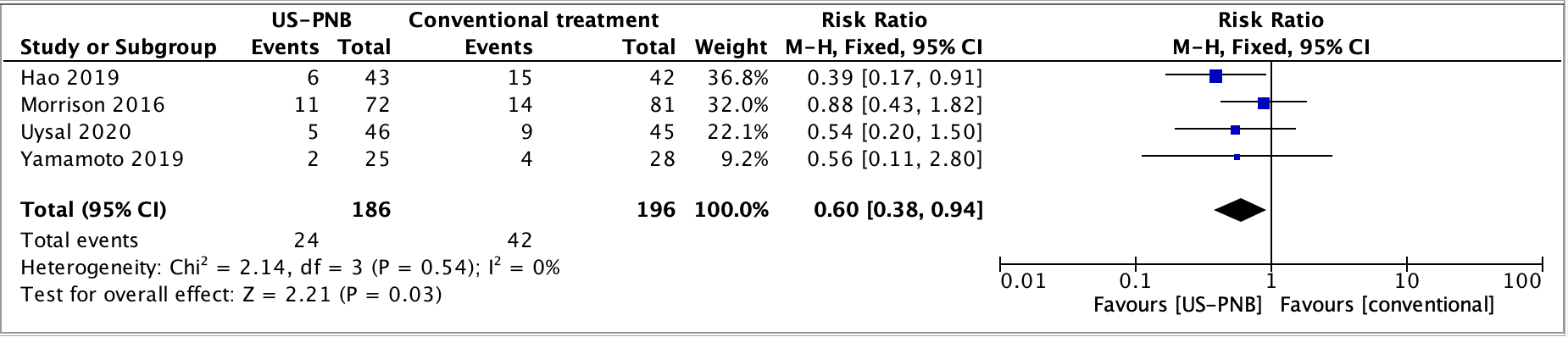


*Forest plot of prevalence of delirium after preoperative ultrasound guided peripheral nerve blocks compared to systemic analgesia in hip fracture patients.*

*US-PNB, ultrasound-guided peripheral nerve block.*

**Supplementary Figure 4. Length of stay**


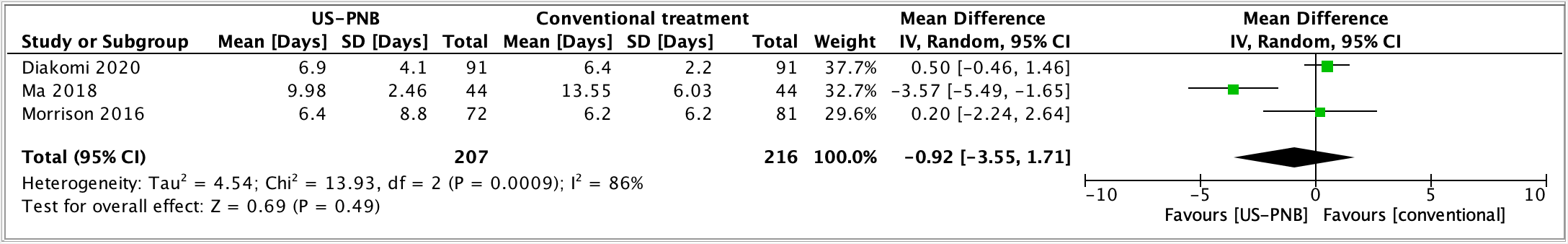


*Forest plot of length of stay after preoperative ultrasound guided peripheral nerve blocks compared to systemic analgesia in hip fracture patients. Mean and SD are presented in number of days.*

*US-PNB, ultrasound-guided peripheral nerve block.*

**Supplementary Figure 5. Mortality**


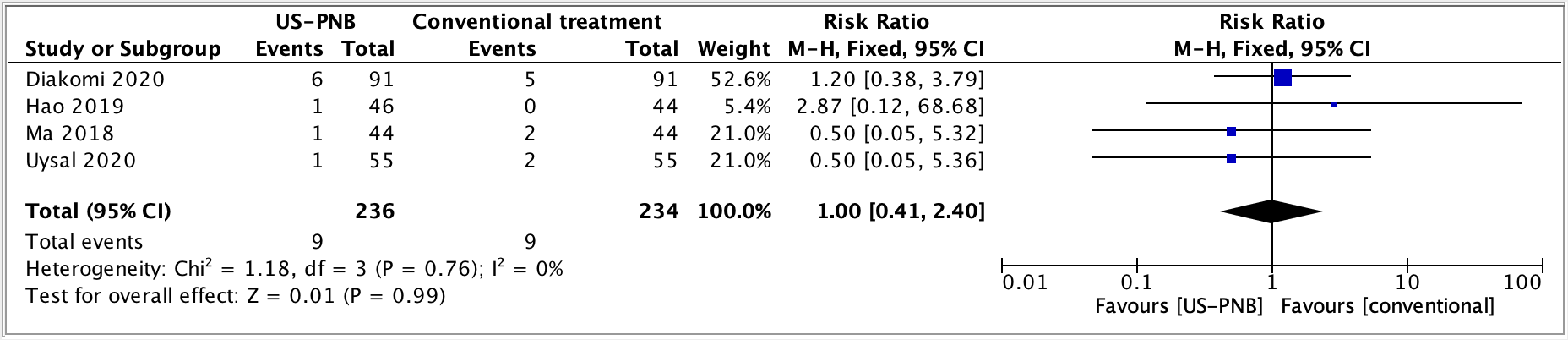


*Forest plot of mortality after preoperative ultrasound guided peripheral nerve blocks compared to systemic analgesia in hip fracture patients.*

*US-PNB, ultrasound-guided peripheral nerve block.*
