# Supplementary material for: Ultrasound-guided peripheral nerve blocks for preoperative pain management in hip fractures: a systematic review
Source: BMC Anesthesiol. 2022 Jun 21;22:192. doi: 10.1186/s12871-022-01720-7 (PMC9210678; doi:10.1186/s12871-022-01720-7)
Supplement: Supplementary file 2 — Additional file 2. Search strategy. [file 12871_2022_1720_MOESM2_ESM.docx]

**Additional file 2: Search strategy**

Concept 1 (nerve blocks):

((((((((((((((((((((((((((((((((("Nerve Block"[Mesh]) OR (Nerve blocks)) OR (peripheral nerve block)) OR (peripheral nerve blocks)) OR (Peripheral nerve blockade)) OR (Nerve blockade)) OR (Nerve blockades)) OR (Nervous blockade)) OR (PNB)) OR (fascia iliaca compartment block)) OR (fascia iliaca compartment blocks)) OR (fascia iliaca block)) OR (fascia iliaca blocks)) OR (fascia iliac compartment block)) OR (fascia iliac compartment blocks)) OR (fascia iliac block)) OR (fascia iliac blocks)) OR (femoral nerve block)) OR (femoral nerve blocks)) OR (3-in-1 block)) OR (3-in-1 blocks)) OR ("three in one" block)) OR ("three in one" blocks)) OR (three-in-one block)) OR (three-in-one blocks)) OR (PENG)) OR (PENG block)) OR (PENG blocks)) OR (pericapsular nerve group block)) OR (pericapsular nerve group blocks)) OR (Quadratus Lumborum block)) OR (Quadratus Lumborum blocks)) OR (regional anesthesia))

Concept 2 (hip fractures):

((((((((((((((((((((("Hip Fractures"[Mesh]) OR (Hip Fracture)) OR (Femur head fracture)) OR (Femur head fractures)) OR (Femoral head fracture)) OR (Femoral head fractures)) OR (Femur neck fracture)) OR (Femur neck fractures)) OR (Femoral neck fracture)) OR (Femoral neck fractures)) OR (Trochanteric fracture)) OR (Trochanteric fractures)) OR (Subtrochanteric fracture)) OR (Subtrochanteric fractures)) OR (Intertrochanteric fracture)) OR (Intertrochanteric fractures)) OR (Pertrochanteric fracture)) OR (Pertrochanteric fractures)) OR (Intracapsular fracture)) OR (Intracapsular fractures)) OR (Extracapsular fracture)) OR (Extracapsular fractures).
